# A Study on Damage of T800 Carbon Fiber/Epoxy Composites under In-Plane Shear Using Acoustic Emission and Digital Image Correlation

**DOI:** 10.3390/polym15214319

**Published:** 2023-11-03

**Authors:** Zikai Lin, Changheng Shi, Xiaochu Huang, Can Tang, Ye Yuan

**Affiliations:** 1 Shenzhong Link Management Center, Zhongshan 528400, China; 2 Faculty of Civil Engineering and Mechanics, Jiangsu University, Zhenjiang 212013, China; chshi2022@163.com; 3 College of Civil Science and Engineering, Yangzhou University, Yangzhou 225127, China; 4 School of Electrical and Information Engineering, Jiangsu University, Zhenjiang 212013, China

**Keywords:** in-plane shear, acoustic emission, digital image correlation, factor analysis, Fuzzy C-Means

## Abstract

In addition to measuring the strain, stress, and Young’s modulus of materials through tension and compression, in-plane shear modulus measurement is also an important part of parameter testing of composites. Tensile testing of ±45° composite laminates is an economical and effective method for measuring in-plane shear strength. In this paper, the in-plane shear modulus of T800 carbon fiber/epoxy composites were measured through tensile tests of ±45° composite laminates, and acoustic emission (AE) was used to characterize the damage of laminates under in-plane shear loading. Factor analysis (FA) on acoustic emission parameters was performed and the reconstructed factor scores were clustered to obtain three damage patterns. Finally, the development and evolution of the three damage patterns were characterized based on the cumulative hits of acoustic emission. The maximum bearing capacity of the laminated plate is about 17.54 kN, and the average in-plane shear modulus is 5.42 GPa. The damage modes of laminates under in-plane shear behavior were divided into three types: matrix cracking, delamination and fiber/matrix interface debonding, and fiber fracture. The characteristic parameter analysis of AE showed that the damage energy under in-plane shear is relatively low, mostly below 2000 mV × ms, and the frequency is dispersed between 150–350 kHz.

## 1. Introduction

Fiber-reinforced polymer materials have broad application prospects in engineering. In the aerospace field [1,2,3,4,5], components such as aircraft engine turbine blades and tail wings require materials with sufficient shear strength to ensure the stability and maneuverability of the aircraft. There are generally three methods for testing the in-plane shear strength of materials: the torsion method; the tensile method such as ±45° tensile test; and the shear method such as the track shear test or V-shaped open-track shear test [6,7,8]. The thin-walled tube torsion method has high accuracy while ±45° tensile test has good practicality. Meanwhile, when structural components are subjected to external loads, internal damage may cause structural deformation and decrease in mechanical properties. Consequently, realizing and understanding the damage evolution process of materials under in-plane shear behavior is crucial for structural health monitoring.

There are usually two ways to conduct damage assessment. One type of active technology requires external sources such as thermal imaging [9,10] and eddy current [11], while the other type of passive technology requires sources inside the material. AE has unique advantages in damage localization and assessment. As a passive technology, it cannot directly measure material damage, but rather analyzes the correlation between hidden information in the signal and the observed structural state by collecting signal responses. Various AE parameters were extracted to evaluate the degree of material damage under external forces. Qu et al. [12] used AE energy and peak frequency parameters to evaluate the damage of C/SiC composite materials under shear monotonic loading. Rubio-González et al. [13] compared the damage characteristics of glass fiber/epoxy resin laminates with/without carbon nanotubes under three-point bending state through AE amplitude and count, and verified the coordination of AE and other technologies in studying composite material damage. Liu et al. [14] studied the damage behavior of several lattice-reinforced concretes under three-point bending using peak frequency, energy, and amplitude of AE signal. AE can not only characterize damage under quasi-static testing, but can also be used for dynamic damage characterization such as impact [15] and fatigue [16,17].

In addition to characterizing damage characteristics, the damage patterns of materials can also be identified by clustering appropriate AE parameters [16,18,19,20,21,22,23,24,25]. Mi et al. [18] compared the impact of compilation methods on the damage of fiber–resin composite materials by clustering the amplitude, energy count, and duration of AE parameters. Azadi et al. [20] studied the damage mode characteristics of carbon/epoxy resin laminates under tension at different loading rates by performing clustering on the three types of acoustic emissions including average frequency. Özaslan et al. [21] studied the damage development of composite plates with a single hole or two holes with different orientations relative to the load direction, and obtained the damage modes in both cases using AE and DIC. Andraju et al. [23] conducted tensile tests on carbon-fiber-reinforced composite laminates with different fiber stacking directions and orders, and the peak frequency and amplitude range of damage in the laminates with different stacking methods were compared. Sawan et al. [24] studied damage types and development modes corresponding to different clusters of epoxy resin composite material specimens under tension and compression by clustering five AE time-domain characteristic signals. Xu et al. [25] analyzed the mechanical behavior response and damage mode of joints under humid and hot conditions using time-frequency domain analysis and clustering analysis of four AE characteristic parameters. However, for the clustering of polymer materials, existing research tends to choose two or a small amount of AE signals, which may result in a lack of information contained in the acoustic emission signals. It is possible to consider recombining multiple AE feature parameters to identify the damage mode of the material while preserving information in dimensionality reduction. Various clustering methods have been developed, such as the k-Means method [16,24,26], Fuzzy C-Means [20,27], and Self-Organizing Map [28]. Different clustering methods are suitable for different classification scenarios; k-Means is suitable for simple classification. However, it does not perform well in handling classification tasks with high mixing and is prone to falling into local optima, while Fuzzy C-Means specifies the membership degree of sample classes between (0, 1) instead of assigning them to specific categories making it suitable for complex classification tasks. Simultaneously, to evaluate the clustering results, multiple indicators can be used for clustering evaluation such as the Silhouette Index, Dunn Index, Davies–Bouldin Index, and Rand Index [16,19,23,29]. 

Due to the fact that AE cannot directly measure the damage mode of materials, it is often combined with other damage assessment methods. In many studies [14,30,31,32,33,34], the DIC method had been used as another complementary detection method in conjunction with AE detection of material damage, with non-contact methods used to measure the surface strain and deformation fields of objects. Although DIC cannot obtain internal deformation and strain information of materials, it can still provide a reference for evaluating material damage.

Existing studies tends to choose two parameters—peak frequency and amplitude not affected by damage for clustering, or extract a small number of parameters based on principal component analysis. However, fewer parameters can lead to the loss of information of AE signal. Therefore, the focus of this paper is using factor analysis to reorganize the original AE parameters into three factors and cluster them. Due to the difficulty in identifying the membership categories of the reorganized factors, the FCM clustering method was chosen, and the number of clusters was evaluated. The results indicate that clustering after factor recombination of eight AE characteristic parameters can obtain the damage mode of the laminates while retaining most of the AE signal information. DIC can provide reference for damage analysis by conducting strain detection on the surface of the laminates.

## 2. Experimental Details

The T800 carbon fiber/epoxy-resin-reinforced polymer laminates were produced by the AECC Beijing Institute of Aeronautical Materials (Beijing, China). The laying angle is ±45°, and each layer of carbon fiber has a thickness of 0.1 mm, totaling 20 layers. The laminates were formed using a hot-pressing process, maintaining a vacuum during the pre-treatment and curing processes. After the pre-impregnated material was laid, the pre-formed parts were pressurized at 320–330 °C, and the curing temperature was set between 330–380 °C after pressurization. Curing stress was reduced through multi temperature layered curing, with a maximum curing temperature of 380 °C and a curing time of 1 h. Five specimens were cut according to the ASTM D3518 standard, with the size of 250 mm × 25 mm × 2 mm. The strain pattern was pasted on the one side before the experiments and speckles were sprayed on the opposite side surface of the specimens.

**Figure 1:**
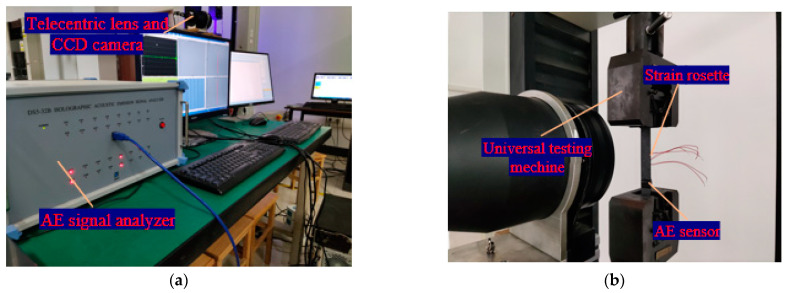
The experimental setup. (**a**) The AE and DIC experimental device. (**b**) Specimen installation diagram.

Figure 1 shows the experimental setup of AE and image data acquisition equipment. The loading equipment is a DNS electronic universal testing machine with an accuracy of ±0.5% of the numerical display, with a maximum experimental force of 100 kN. According to ASTM D3518, the in-plane shear test should be completed within 1–10 min. As a result, the loading rate should be set to 4 mm/min according to prior experiments. 

Throughout the in-plane shear experimental process, an image acquisition device was used to capture images, and the image collection frequency was set to 15 images per second. The image acquisition device was composed of a five-megapixel CCD camera, a 0.072 magnification telecentric lens, and a light source. Concurrently, the AE signals were collected through the DS2-8A AE signal analyzer produced by Ruandao (Abu Dhabi, United Arab Emirates) including a 40 DB integrated preamplifier and a cylindrical probe with a diameter of 8 mm, which was fastened to the specimen using coupling agent and insulation tape. The experimental acquisition accuracy was 16 bits, with the rate of 3 and threshold set to 30 mV. The strain rosettes were glued to the center of the specimens with a resistance value of 120 Ω and a sensitivity coefficient of 2 ± 1%. It adopted a 1/4 bridge connection method and temperature compensation. The sampling frequency of strain data is 1 Hz. A lead breaking test was tried out before conducting an in-plane shear experiment on each specimen.

## 3. Results and Discussion

### 3.1. Tangential Shear Modulus Calculation and Parameter Analysis of AE Signals

**Figure 2:**
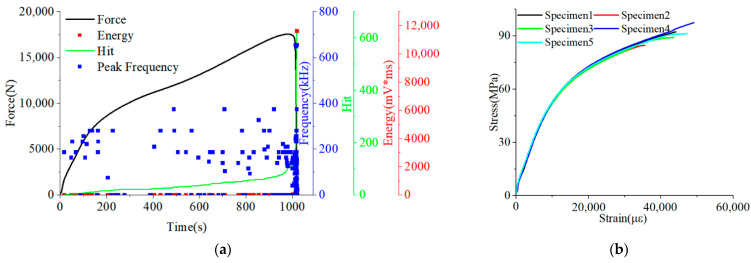
The AE time-domain signals and stress-strain curves of specimens. (**a**) The AE time-domain signals of specimen 1. (**b**) The stress-strain curve of specimens.

Figure 2a shows the force-time curve with the time-domain composition curves: energy, hit, and peak frequency extracted from the obtained AE signals of specimen 1, while Figure 2b shows in-plane shear stress-strain curves of specimens. In the AE and force time-domain signal graph, the tensile process was divided into three stages based on the trend of force over time. In stage I, the response curve between force and time is linear elastic, with relatively few AE signals and a peak frequency basically below 200 kHz. In stage II, the force-time curve begins to exhibit a non-linear response, with a decrease in the rate of force growth over time and an increase in AE signals at medium to high peak frequencies, while the acoustic emission signals are relatively sparse. In stage III, the growth rate of force increases with time and reaches its peak; afterwards, it briefly decreases and the specimen underwent fracture and failure. In addition, at the failure time of stage III, a sudden change in the AE energy signal can be clearly observed, with an energy value of 12,000 mV × ms. This is because the fracture of the sample released a significant amount of energy. Other energy points were lower, with energy values not exceeding 200 mV × ms. At the same time, the AE hit also showed a straight upward trend due to the generation of more AE signals caused by specimen fracture.

In Figure 2b stress-strain curve exhibits a linear response during the initial stage. After the strain reaches 2000 µε, the stress trend of the sample is relatively stable. At this stage, a strain point was selected as the starting point, then the shear strain was increased by 4000 ± 200 µε as the endpoint to calculate the tangential shear modulus. The calculation results are shown in Table 1. The average value of tangential shear modulus is 5.42 GPa and the dispersion coefficient value is 0.95%.

### 3.2. DIC Analysis

The longitudinal strain and displacement were calculated through setting the software spectrum to 16, and the area from the center of the sample to the failure range was selected. The sample image at 527 s was selected as the reference image and the subsequent images were processed. The image processing results before 527 s can refer to the trend of displacement and strain changes at 711 s and 991 s.

**Figure 3:**
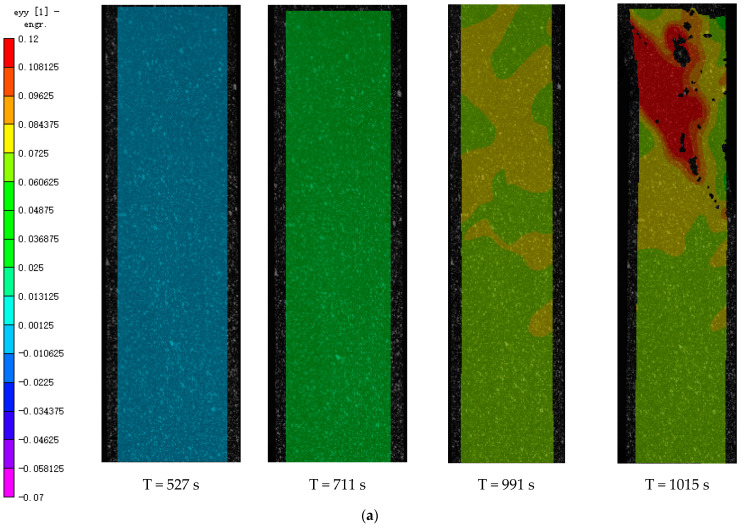

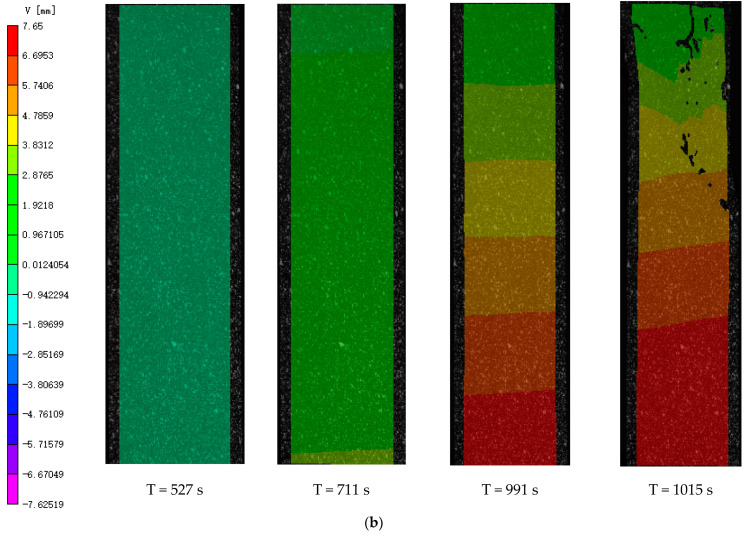
Results of DIC. (**a**) Longitudinal strain at different times. (**b**) Longitudinal displacement at different times.

As shown in Figure 3a, when the strain is relatively small, the longitudinal strain is more uniform. As the strain increases, higher strain regions gradually appear. As the specimen approaches failure, necking and strain concentration occur in the failure area. In addition, as shown in Figure 3b, the longitudinal displacement of the sample before failure presents a stepped shape. Due to the fixed clamp at the upper end of the testing machine during stretching, the displacement shows an increasing trend from top to bottom, and the displacement contour line is basically horizontal. During failure, the deformation of the displacement contour is more pronounced in the upper part of the specimen.

### 3.3. Factor Analysis

The damage modes of carbon fiber laminates can be analyzed by clustering the AE characteristic parameters. In this regard, existing studies are mostly based on peak frequency and amplitude. However, a small number of AE characteristic parameters cannot fully characterize the characteristics of AE signals, and excessive AE characteristic parameters will increase the difficulty of analysis. Therefore, the AE parameters were first dimensionally reduced through FA before implementing clustering, and new components that contain most of the AE signal information were obtained through factor recombination.

The eight characteristic parameters of amplitude, duration, rise time, ring counts, rise counts, energy, center frequency, and peak frequency reflecting the characteristics of AE signals in the time-frequency domain were extracted for FA analysis. Parameters data were standardized to reduce the impact of data magnitude. 

Table 2 showed the Pearson correlation between eight parameters by calculating Pearson coefficient and significance, where significance less than 0.01 indicates a very significant correlation and the data are marked with ‘**’, and significance less than 0.05 indicates a significant correlation and the data are marked with ‘*’. The results indicate that there is a strong correlation between the eight AE parameters; therefore, FA can be performed.

**Figure 4:**
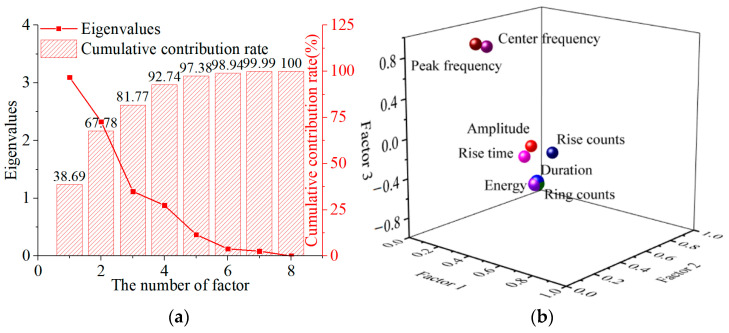
The extraction results of FA. (**a**) Eigenvalues and cumulative contribution rate of FA. (**b**) The spatial distribution of factor loading.

Extracting the number of factors is a process of comprehensive selection, usually determined by variance contribution rate and eigenvalues to determine the number of factors extracted by FA. The variance contribution rate and eigenvalues demonstrate the ability of factors to interpret raw AE parameter information, and the larger their values, the stronger their explanatory power. At the same time, the component matrix was calculated, which displays the relationship between factors and original parameters. By rotating the component matrix, the relationship between factors and original parameters can be better explained. The factor matrix is shown in Table 2, the cumulative contribution rate and eigenvalues are shown in Figure 4a, and the factor loading diagram of the component matrix is shown in Figure 4b.

The number of factors can be selected based on different situations. In this analysis, the criterion for selecting is that the eigenvalues are greater than 1. From Figure 4a, it can be seen that the first three factors meet the selection requirements and have a cumulative contribution rate greater than 80%. Therefore, the number of factors was determined to be 3 in this analysis. As shown in Table 3, factor loading with the three parameters duration, ring counts, and energy are greater than 0.95, and they are positive to Factor 1. Similarly, amplitude, rise time, and rise count have a positive effect to Factor 2 while duration, ring count, and energy are negative to Factor 2. Center frequency and peak frequency have a positive effect on Factor 3. Factor 1 and Factor 2 reflect the time domain characteristics of AE signals and Factor 3 representative frequency domain characteristics. 

The score coefficients of the three components were calculated as shown in Table 4 and the value of Factor 1, 2, and 3 were obtained on this basis. As for Factor 1, the score is calculated as follows:

$$S_{1}=0.063x_{1}+0.321x_{2}+\ldots +0.021x_{8}$$

where $S_{1}$ represents the score of Factor 1, $x_{1}$, $x_{2}$,…, $x_{8}$ represents the value of amplitude, duration… peak frequency of every AE events.

### 3.4. Fuzzy C-Means

To further explain the relationship between factors and clusters, score statistics were performed on the clustering results. The FCM algorithm is a fuzzy partitioning-based algorithm that maximizes the similarity of the same cluster and minimizes the similarity of different clusters by calculating the membership of different categories of sample points. 

Assuming the number of clusters in the sample set $X=\left\{x_{1},x_{2},\ldots ,x_{n}\right\}$ is *c*, and the FCM objective function is as follows:

(1) $$J(U,V)=\sum_{i=1}^{c}\sum_{j=1}^{n}{\left(u_{\mathrm{ij}}\right)}^{m}d_{ij}^{2}$$

$$d_{ij}^{2}={\left\|c_{i}-x_{j}\right\|}^{2}$$

where $u_{ij}$ is membership matrix value between (0, 1); *m* is the fuzzy weighted index; $d_{ij}$ is the Euclidean distance between sample and cluster center; *U* is the fuzzy membership set.

Set the constraint condition that the sum of all sample membership degrees is 1:

(2) $$\sum_{i=1}^{c}u_{ij}=1,\forall j=1,\ldots ,n$$

To minimize the objective function, the Lagrange method is used to solve the Lagrange function, which is as follows:

(3) $$J(U,c_{1},\ldots ,c_{c},\lambda_{1},\ldots ,\lambda_{n})=J(U,c_{1},\ldots ,c_{c})+\sum_{j=1}^{n}\lambda_{j}(\sum_{i=1}^{c}u_{ij}-1)=\sum_{i=1}^{c}\sum_{j}^{n}u_{ij}d_{ij}^{2}+\sum_{j=1}^{n}\lambda_{j}(\sum_{i=1}^{c}u_{ij}-1)$$

The clustering center and membership degree were obtained by solving the following:

(4) $$c_{i}=\frac{\sum_{j=1}^{n}u_{ij}x_{j}}{\sum_{j=1}^{n}u_{ij}}$$

(5) $$u_{ij}=\frac{1}{\sum_{k=1}^{c}{\left(\frac{d_{ij}}{d_{kj}}\right)}^{2/(m-1)}}$$

When clustering data, determine the clustering center and membership matrix by following these steps:

Step 1: Initialize the membership matrix *U* and randomly assign values to (0, 1), satisfying the constraint conditions;

Step 2: Calculate cluster center;

Step 3: Calculate the objective function. If it is less than the set threshold or if the difference between adjacent calculations is less than the threshold, stop;

Step 4: Bring in the membership degree calculation formula to recalculate the *U* and return to step 2.

**Figure 5:**
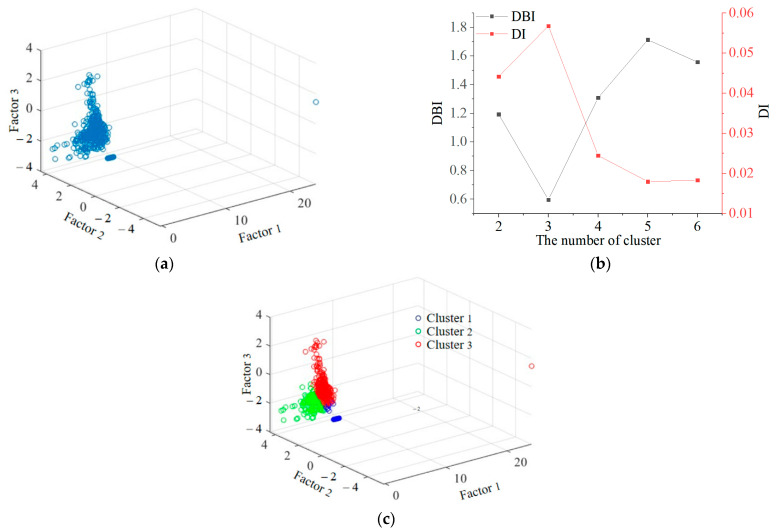
FCM results on specimen 1. (**a**) Spatial distribution of three factors. (**b**) DBI and DI of FCM. (**c**) FCM three-dimensional diagram result on the scores of factors.

Figure 5a shows the spatial distribution of the three factors; it can be seen that each sample point does not have a specific category, making it suitable to use the fuzzy clustering method. Before clustering, it is first necessary to determine the appropriate number of clusters. The Dunn index (DI) and Davies–Douldin index (DBI) were applied to evaluate the number of clusters. DI represents the ratio of the minimum distance between any two clusters to the maximum distance between two sample points in any cluster. DBI evaluates the number of clusters by calculating the similarity between each cluster and other coarse values. The larger the DI, the better the clustering effect, while the smaller the DBI, the better the clustering effect. For carbon fiber composite materials, the damage modes are generally divided into four categories. The corresponding evaluation indicators for the number of 2–6 clusters were calculated as shown in Figure 5b. It can be determined that the optimal number of clusters is 3. The clustering results are shown in Figure 5c.

Score intervals for the corresponding factors of three clusters were obtained. The statistical results were summarized in Table 5. There are a total of 619 AE data sample points, with 134 in Cluster 1, 176 in Cluster 2, and 309 in Cluster 3. The larger the absolute value of the numerical value, the greater the impact of the factor on the cluster. The positive and negative values represent the positive and negative effects of the factor.

**Figure 6:**
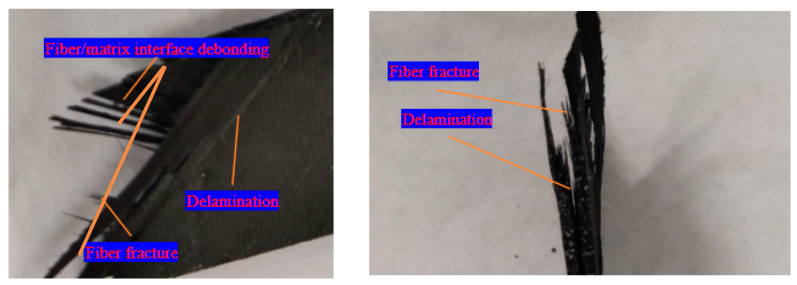
Fracture image of specimen 1.

Overall, Cluster 1 has the smallest and mostly negative cluster score values on the three factors, while Cluster 3 has the highest cluster score on the three factors. Therefore, the AE parameters of Cluster 1 have relatively low intensities, exhibiting characteristics such as low amplitude, low duration, and low frequency. In addition, three types of damage including fiber breakage can be identified from fracture images, as shown in Figure 6. Therefore, Cluster 1 is matrix cracking. Cluster 3 has the highest intensity of AE parameters, exhibiting high amplitude, high energy, and high frequency characteristics. It is worth noting that the upper limit of the score interval for Factor 1 in Cluster 3 is 24.06, which is significantly higher than the upper limit of the interval for the other two clusters. According to the previous section on factor analysis, energy has a strong contribution to Factor 1. It can be inferred that Cluster 3 includes the AE event of enormous energy generated by the failure and fracture of the specimen. Therefore, Cluster 3 is a fiber fracture. The AE parameter properties of Cluster 2 are between the two; therefore, Cluster 2 is delamination and fiber/matrix interface debonding. 

**Figure 7:**
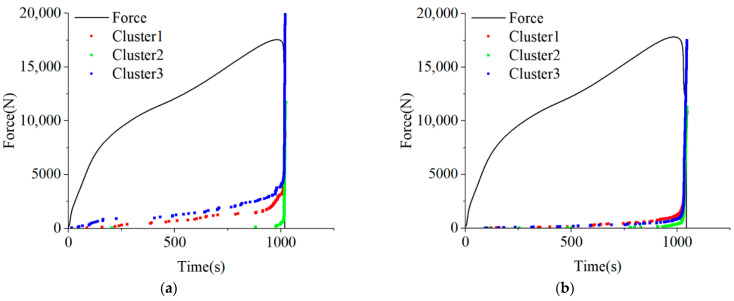

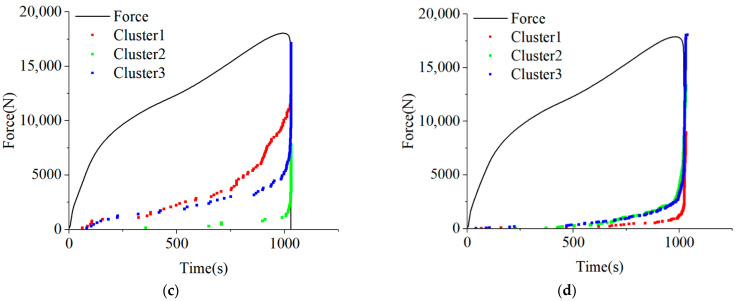
The distribution of the cluster. (**a**) Specimen 1. (**b**) Specimen 2. (**c**) Specimen 3. (**d**) Specimen 4.

Finally, based on the clustering results, the AE hits were divided to obtain the development and evolution trends of each mode. The evolution of damage modes is shown in Figure 7. The results indicate that the development of each damage mode is similar. Before failure, the development of various damages is relatively slow. As the failure approaches, the trend of damage evolution turns and randomly increases rapidly. Overall, there are more fiber fractures during in-plane shear, occupying a dominant position. The minimum number of delamination and fiber/matrix interface debonding may be related to the properties of the material.

## 4. Conclusions

This paper investigated the in-plane shear failure mode and damage evolution behavior of T800 carbon fiber/epoxy composite materials through AE and DIC. FA was used to evaluate the relevance between AE parameters. It is difficult to distinguish the membership categories of factors after restructuring the acoustic emission feature parameters, and the Fuzzy C-Means cluster algorithm was used. The characteristic parameter analysis of AE showed that the damage energy under in-plane shear is relatively low, mostly below 2000 mV × ms, and the frequency is dispersed between 150–350 kHz. The fracture of laminates is in a sudden and the energy at fracture is close to 12,000 mV × ms, with frequency concentrated between 0–200 kHz. The AE hit increased slowly before fracture, reaching a cumulative count of 100 at 1000 s, and then showed a straight upward trend, increasing to 600 in a short period of time, which indicated that the accumulation of damage in laminates is slow and the damage mainly accumulates at the moment of fracture. The FA indicated that there is a high correlation between the AE characteristic parameters under in-plane shear, and the development and evolution of three damage modes were obtained through an unsupervised Fuzzy C-Means cluster algorithm. The clustering effect is better for Cluster 3, corresponding to matrix cracking, delamination, fiber/matrix interface debonding, and fiber fracture. Cluster 3 dominates the in-plane shear process, with more events than Cluster 1 and 2. Overall, the trends of development of the three damage patterns were similar. The DIC analysis results indicate that the strain and deformation on the surface of the specimen are relatively uniform, and there is a significant strain concentration when the specimen is near failure. This can be verified by the flat growth trend of the three identified damage modes before 1000 s and the rapid upward trend around 1000 s. DIC can be well combined with AE to evaluate the damage of specimens. There are also some shortcomings in this work that require further research, such as the impact of specimen damage on the transmission of AE signals in the specimen. However, the result of the study can still refer to the establishment of structural health monitoring standards, evaluations, and related databases.

## Figures and Tables

**Table 1:** Tangential shear modulus of specimens.

Number of specimens	1	2	3	4	5
Value (GPa)	5.31	5.23	5.69	5.35	5.48

**Table 2:** Pearson coefficient of AE parameters of specimen 1.

	Amplitude	Duration	Rise Time	Ring Counts	Rise Counts	Energy	Center Frequency	Peak Frequency
Amplitude	1	0.051	0.243 **	0.064	0.618 **	0.053	0.105 **	0.065
Duration		1	0.119 **	0.994 **	0.087 *	0.993 **	0.005	0.038
Rise time			1	0.106 **	0.529 **	0.099 *	0.091 *	−0.015
Ring counts				1	0.101 *	0.999 **	−0.002	0.022
Rise counts					1	0.091 *	0.110 **	0.048
Energy						1	−0.006	0.016
Center frequency							1	0.683 **
Peak frequency								1

*—significance less than 0.05 indicates a significant correlation; **—significance less than 0.01 indicates a very significant correlation.

**Table 3:** The factor loading of AE signal parameters.

	Factor 1	Factor 2	Factor 3
Amplitude	0.192	0.694	−0.300
Duration	0.980	−0.178	0.059
Rise time	0.246	0.580	−0.340
Ring counts	0.982	−0.178	0.045
Rise counts	0.259	0.783	−0.384
Energy	0.979	−0.189	0.045
Center frequency	0.055	0.514	0.756
Peak frequency	0.065	0.423	0.815

**Table 4:** The score coefficient matrix of factors.

	Factor 1	Factor 2	Factor 3
Amplitude	0.063	0.352	−0.188
Duration	0.321	−0.090	0.037
Rise time	0.080	0.294	−0.213
Ring counts	0.321	−0.090	0.028
Rise counts	0.085	0.397	−0.241
Energy	0.321	−0.096	0.028
Center frequency	0.018	0.261	0.473
Peak frequency	0.021	0.214	0.510

**Table 5:** Results of cluster statistics.

	Factor 1	Factor 2	Factor 3
Cluster 1	−0.333, 0.079	−1.544, −0.260	−1.122, −0.040
Cluster 2	−0.076, 1.542	−0.086, 4.516	−3.230, 0.514
Cluster 3	−0.245, 24.061	−5.403, 1.739	−0.384, 3.794

## Data Availability

Data will be made available on request.
